# A Doppler Range Compensation for Step-Frequency Continuous-Wave Radar for Detecting Small UAV

**DOI:** 10.3390/s19061331

**Published:** 2019-03-16

**Authors:** Massimiliano Pieraccini, Lapo Miccinesi, Neda Rojhani

**Affiliations:** Department of Information Engineering, University of Florence, via Santa Marta, 3 50139 Firenze, Italy; lapo.miccinesi@unifi.it (L.M.); neda.rojhani@unifi.it (N.R.)

**Keywords:** continuous wave, Doppler, radar, UAV detection

## Abstract

Step-frequency continuous-wave (SFCW) modulation can have a role in the detection of small unmanned aerial vehicles (UAV) at short range (less than 1–2 km). In this paper, the theory of SFCW range detection is reviewed, and a specific method for correcting the possible range shift due to the Doppler effect is devised. The proposed method was tested in a controlled experimental set-up, where a free-falling target (i.e., a corner reflector) was correctly detected by an SFCW radar. This method was finally applied in field for short-range detection of a small UAV.

## 1. Introduction

The increasing number of flying small unmanned aerial vehicles (UAV) poses a serious threat for the safety of flights, as well as for privacy. A critical issue is to prevent UAVs from being used for terrorist attacks, espionage, or other malicious activities against sites with critical infrastructures [1].

Radars can appear as the technology of choice for detecting them, but standard air defense is ill-prepared for UAV detection: the radar cross section of a small UAV can be smaller than 0.03 m^2^ [2], and UAVs are low-velocity aircrafts flying in a high-clutter environment.

Step-frequency continuous-wave (SFCW) radars are widely used in short-range applications like level gauge [3,4], ground-penetrating radar [5,6], and ground-based synthetic-aperture radar [7,8]. The unique advantage of SFCW modulation is its high duty cycle (up to 100%) that allows it to obtain the highest signal-to-noise ratio (SNR) [9]. Its limit is related to the duration of each single tone that has to be longer than the duration of flight of the farthest target. As the target should be stationary during the frequency sweep, the SFCW modulation cannot be used for either long-range detection or fast-moving target tracking. Nevertheless, small UAVs operate at short range and low speed, so SFCW radars can have a role in their detection [10].

Usually, SFCW radars detect the range of targets by calculating the inverse fast Fourier transform (IFFT) of the signal gathered in the frequency domain. Unfortunately, when the target is moving (even at relatively low speed), the Doppler effect gives an apparent range shift that has to be compensated.

Although the Doppler effect in frequency modulation (FM) radars has been extensively studied [11], SFCW modulation has received less attention. Nevertheless, since 1999, Gill [12] correctly identified the range shift due to the Doppler effect in step-frequency radars without proposing a specific technique for correcting/compensating it. The standard approach for compensating the Doppler effect is to transmit a positive and a negative sweep in rapid succession [13]. The Doppler shift has an opposite effect in the two sweeps and so it can be removed. A quite different approach has been proposed by Chen et al. [14]. Their idea was to transmit two trains of tones. The pulse repetition interval (PRI) of the second pulse train is two times that of the first. In this way, with suitable processing, it is possible to compensate for the Doppler effect. Both approaches are based on special waveforms different from a pure (i.e., positive) SFCW. The novelty of the technique proposed in this paper is that it can be implemented without a change in waveform. Therefore, it can be applied to any SFCW radar, even if it is not specifically designed for operating with fast-moving targets.

## 2. Materials and Methods

### 2.1. Theoretical Formulation of the Problem 

**Figure 1:**
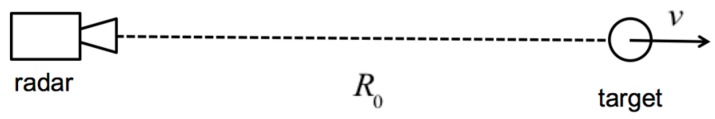
Radar and target moving at speed *v* away from the radar.

We consider a single point target moving at speed *v* in front of an SFCW radar (Figure 1).

**Figure 2:**
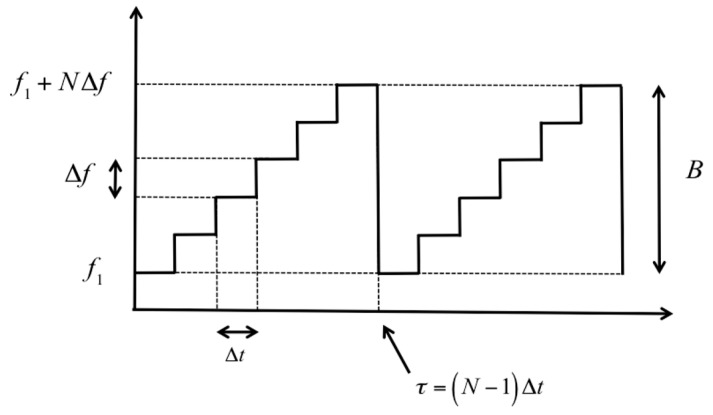
Sweeping of a step frequency continuous wave (SFCW) radar: *f*_1_ is the initial frequency, Δ*f* is the step frequency, *B* is the bandwidth, Δ*t* is the tone duration, *N* is the number of frequencies, and *τ* is the sweep time.

As sketched in Figure 2, the radar sweeps the bandwidth *B* in *N* steps, starting from frequency *f_1_*. The time interval of each tone is Δ*t*, so the time sweep is *τ* = (*N* − 1) Δ*t*. The central frequency is *f_c_* = *f_1_* + *B*/2, and the wavelength *λ* = *c*/*f_c_*, with *c* being the speed of light.

If *v* << *λ*/Δ*t*, we can consider the target stationary during each single tone, and therefore, the signal backscattered by the target at *n*-th frequency can be written as:

(1) $$E_{n}=E_{0}e^{-j\frac{4\pi }{c}\left(f_{1}\;+\;n\Delta f\right)\left(R_{0}\;+\;nv\Delta t\right)}$$

It is worth noticing that this expression is correct only for a short-range operation. It means that the tone duration has to be longer than the time of flight from the farthest target. As an example, a SFCW CWSF radar with a duty cycle of at least 0.5 must respect the following condition:

(2) $$R_{0}<\;\frac{c\Delta t}{4}$$

The trace in range domain is obtained as:

(3) $$I\left(R\right)=\overset{N\;-\;1}{\underset{n\;=\;0}{\sum }}E_{n}e^{-j\frac{4\pi }{c}\left(f_{1}\;+\;n\Delta f\right)R}$$

**Figure 3:**
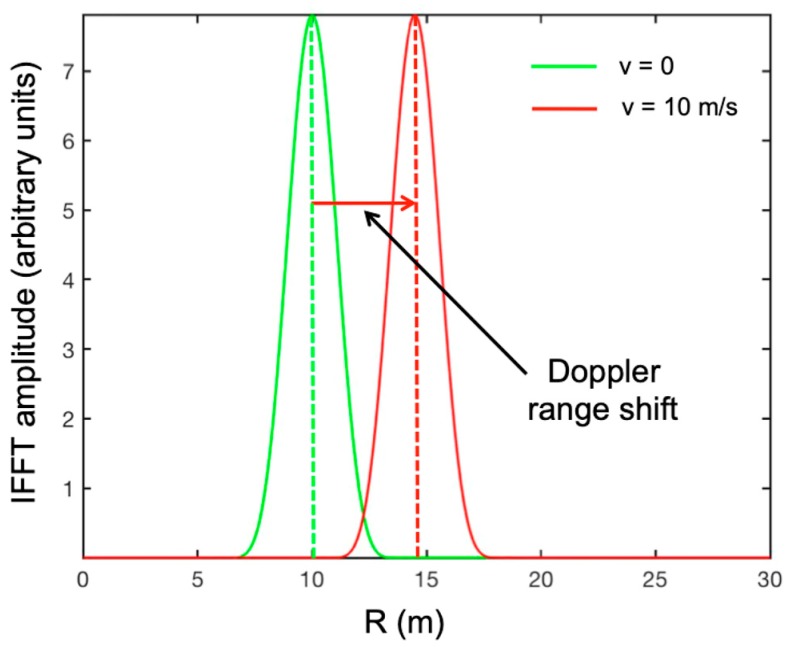
Example of the Doppler range shift. The green curve is the inverse fast Fourier transform (IFFT) amplitude of a static target, the red curve is the IFFT amplitude of a target moving away from the radar (in the radar-target direction) at speed *v* = 10 m/s.

The expression in Equation (3) is mathematically equivalent to the IFFT of the signal: indeed, the information gathered in the frequency domain is transformed in the time/space domain by Equation (3). Unfortunately, the direct application of the IFFT to moving targets gives an incorrect range position. Figure 3 shows an example. The parameters we used were *f_1_* = 16.67 GHz, Δ*f* = 375 kHz, *N* = 400, Δ*t* = 10 μs, *v* = 10 m/s. The green curve is the IFFT amplitude of a static target (*v* = 0 m/s) at *R*_0_ = 10 m. The red curve shows the IFFT amplitude of a target located at *R_0_* = 10 m (*t* = 0) and moving away from the radar (in the radar-target direction) at speed *v* = 10 m/s.

The effective shift during the sweep time *τ* was only 0.04 m, but the Doppler effect gave an apparent forward shift of 4.49 m. This was a detection error that had to be corrected.

With the aim to find an explicit expression for the Doppler range shift, we substituted Equation (1) into Equation (3). By making the following two assumptions:

(4) $$\frac{B}{f_{c}}\ll 1\;(\mathrm{narrow}\;\mathrm{bandwidth})$$

(5) $$\frac{\tau v}{R_{0}}\ll 1\;(\mathrm{far}\;\mathrm{target})$$

with a few algebraic steps, we obtained:

(6) $$I\left(R\right)=E_{0}e^{-j\frac{4\pi }{c}\left(f_{c}\left(R\;-\;R_{0}\right)\;+\;f_{1}\frac{v\tau }{2}\right)}A_{N}\left(x\right)$$

with:

(7) $$A_{N}\left(x\right)=\frac{\sin\left(\frac{N}{2}x\right)}{\sin\left(\frac{x}{2}\right)}$$

(8) $$x=\frac{4\pi }{c}\Delta f\left(R\;-\;\left(R_{0}+\;\frac{f_{1}}{B}v\tau \right)\right)$$

**Figure 4:**
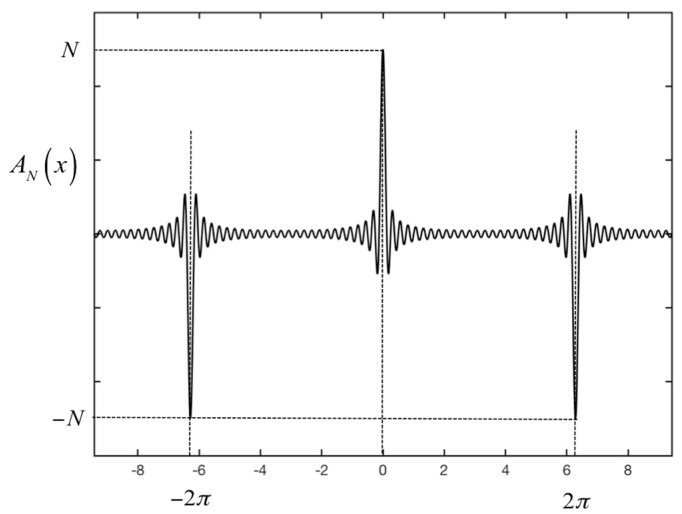
Plot of the mathematical function *A_N_*(*x*).

Figure 4 plots *A_N_*(*x*) as a real function of *x*. Using Equation (8) that links *x* with *R* we noted that |*A_N_*(*x*)| corresponded to a series of peaks at range:

(9) $$R=R_{0}\;+\;\Delta R_{doppler}\;+\;mR_{U}$$

with:

(10) $$\Delta R_{doppler}\;=\;\frac{f_{1}}{B}v\tau$$

(11) $$R_{U}\;=\;\frac{c}{2\Delta f}$$

and *m* natural number.

The physical meaning of Equation (9) is that the apparent range position of the target depends on its speed (i.e., Doppler ambiguity). This problem cannot be solved in general, but if the acceleration of the target respects the following limit

(12) $$\left|\frac{dv}{dt}\right|\;\ll \;\frac{2}{\tau }\left|v\right|,$$

the term *f_1_vτ/*2 inside the argument of the exponential in Equation (6) can be neglected, and the speed can be obtained through interferometry between two subsequent sweeps. This condition is a critical point: it means that this technique works for any speed as long as the speed variations (normalized with respect to speed) are moderate enough. As rule of thumb, the first term in Equation (12) should be lower than one-tenth of the second term. So, by neglecting the term *f_1_vτ*/2 inside the argument of the exponential in Equation (6), the phase difference between two subsequent sweeps at range *R* (close to *R*_0_) is given by:

(13) $$\Delta \phi \left(R\right)\;=\;\frac{4\pi }{c}f_{c}\Delta R_{0}$$

with Δ*R*_0_ being the displacement of the target during the sweep time *τ*. Therefore, the speed can be evaluated as:

(14) $$v\left(R\right)\;=\;\frac{\Delta R_{0}}{\tau }\;=\;\frac{\lambda }{\tau }\frac{\Delta \phi \left(R\right)}{4\pi }$$

On the basis of the theory above, we implemented the following algorithm for compensating for the Doppler effect in SFCW traces.

(1) The gathered data were disposed in a matrix ***E_i_,_k_*** where the *i* index was relative to the frequency, and the *k* index was relative to time (that proceeded at step *τ*).
(2) The matrix was windowed along the *i*-index with a Kaiser window with *β* = 10.
(3) We calculated the IFFT of each column (*i*-index) with a suitable padding factor (*F* = 50) [15]. The number of elements of each column was *N* × *F*, and the new index was *ii*. The range step was *c*/(2*B* × *F*).
(4) We calculated the phase of the product between each column and the complex conjugate of the previous column.
(5) Using Equation (13), we calculated the estimated speed at each range (identified by the index *ii*).
(6) Using Equation (9), we calculated the Doppler range shift and we divided it by the range step. We estimated the integer number *m* closer to that ratio.
(7) The complex value of the IFFT at range *ii* was summed to the value at range *ii* + *m*.
(8) We repeated steps 5–7 for all *ii*.

### 2.2. Simulation

**Figure 5:**
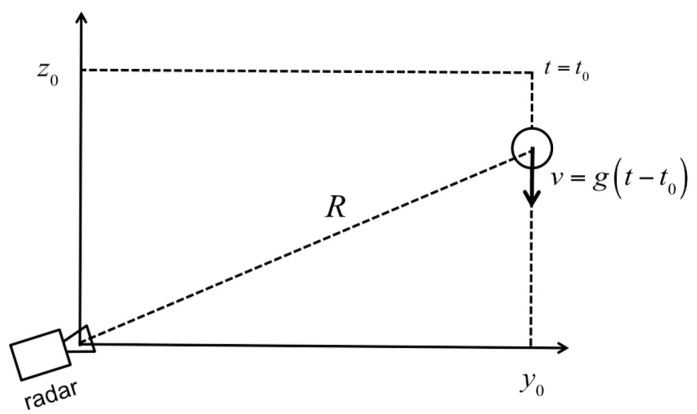
Simulation set-up of the free-falling target.

In order to test the method and the related algorithms, we simulated the experimental set-up sketched in Figure 5. The target for *t* < 0 was in the position (*y*_0_, *z*_0_). The radar was positioned at the origin of the axes. At *t* = 0, the target was free-falling. By neglecting the friction with air, its falling speed was *v* = *g*(*t − t_0_*), with *g* = 9.8 m/s^2^. The simulation parameters were *y*_0_ = 7.39 m, *z*_0_ = 3.0 m, *t*_0_ = 2.05 s, *f*_1_ = 16.67 GHz, Δ*f* = 375 kHz, *N* = 400, Δ*t* = 10 μs, and SNR = 36 dB.

**Figure 6:**
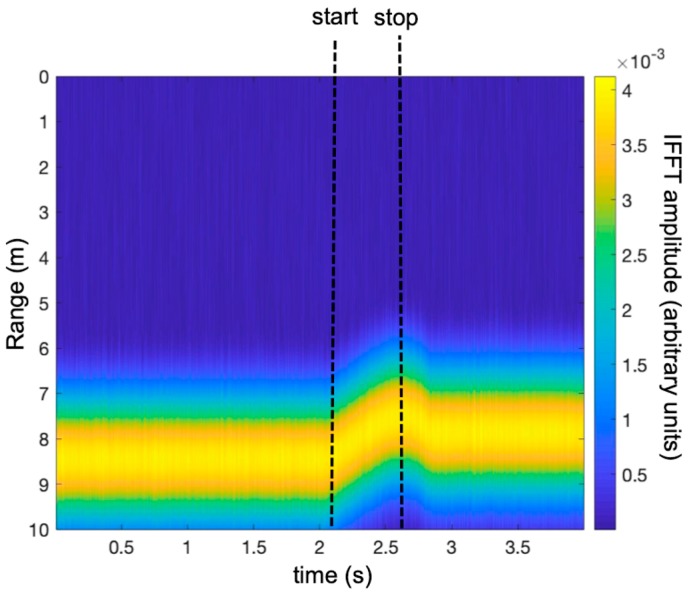
Simulated time-range map of the free-falling target.

Figure 6 shows the map of the IFFT amplitude. The IFFT was calculated along the column of the ***E_i_,_k_*** matrix; so, in the *y*-axis there was the distance from the radar, while in the *x*-axis there was the time (i.e., a trace for each time sweep *τ*).

**Figure 7:**
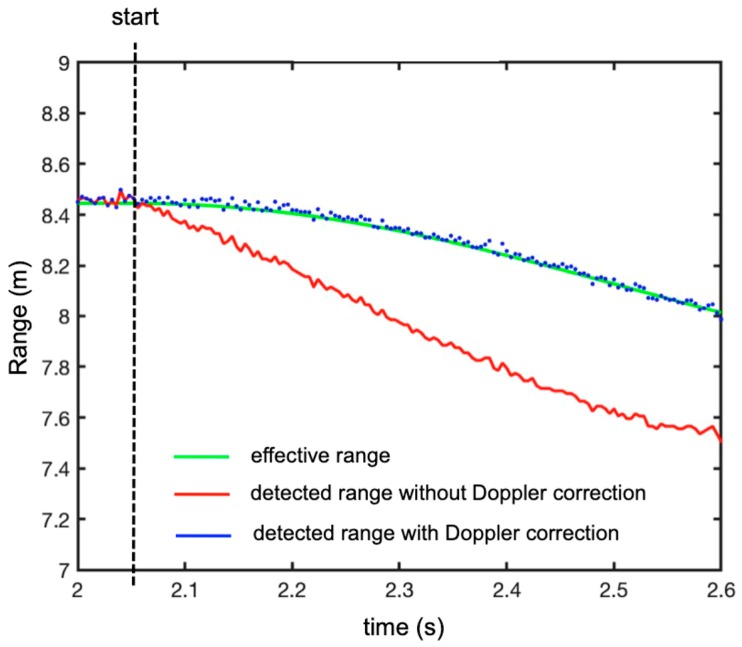
Plot of the peak range in time. The green line is the effective range of the target, the red line is the range of the peaks without the Doppler correction, and the blue dotted line is the range of the peaks with the Doppler correction.

**Figure 8:**
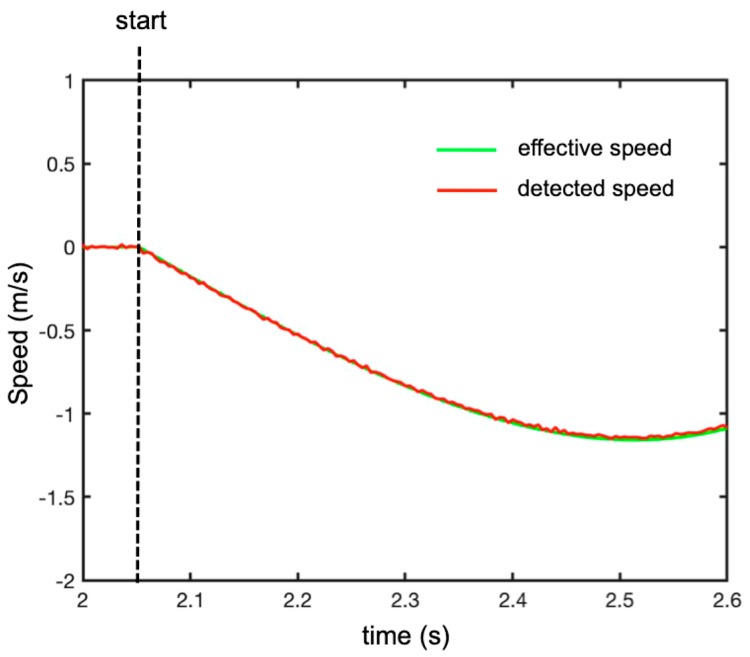
Speed of the falling target (**green line**) and the estimated speed by phase difference between two subsequent sweeps (**red line**).

Figure 7 shows the plot (in red) of the range of the peaks against time. The plot in green is the effective range of the free-falling target. It is evident that the Doppler effect gave a significant error in the estimation of the range. The discrepancy between the apparent and the real position was given by Equation (9). Therefore, it was necessary to evaluate the speed using Equation (13) in order to correct for this bias. Figure 8 shows the speed being evaluated using Equation (13) and the effective speed.

The final step was to apply the range correction to the plot of the peaks in time. The corrected plot is shown in blue in Figure 7. The agreement was very satisfactory.

**Figure 9:**
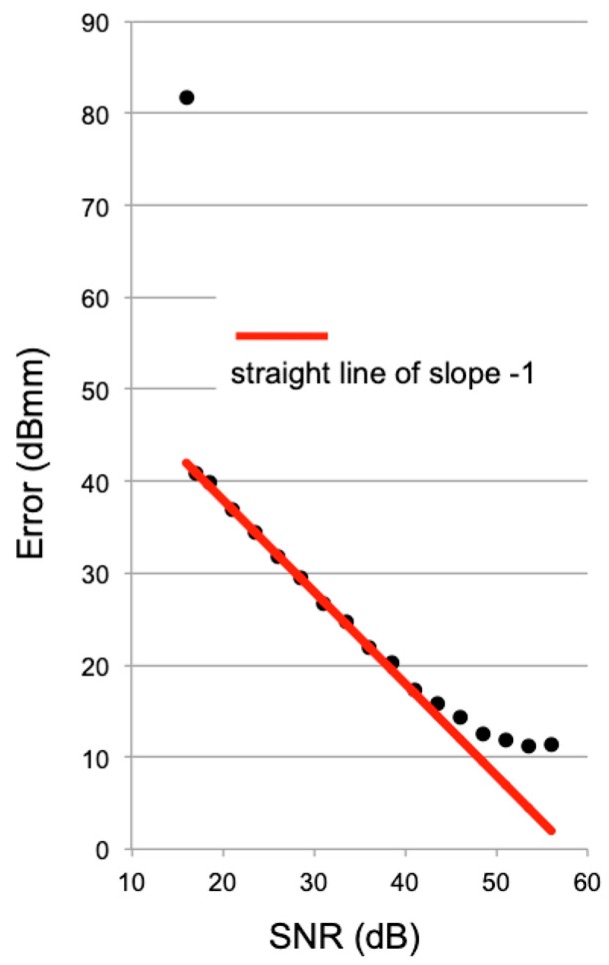
Range of error evaluated for different signal-to-noise ratios (SNR). The red line is an interpolating straight line of slope −1.

In order to evaluate the effect of the white Gaussian noise in the range estimation, we performed simulations with different SNRs. Figure 9 reports the obtained error (evaluated as the standard deviation during a single fall) as a function of the SNR (evaluated as the SNR of one single time trace after the IFFT).

It resulted that when the SNR was lower than 15 dB, the compensating procedure failed, and the error increased dramatically. This threshold effect was probably due to the interferometric procedure used for retrieving the speed that failed when the error overcame λ/2. The error (in logarithmic scale) decreased linearly with the slope −1 between 15 dB and 45 dB of the SNR. This behavior was rather typical when the error was estimated as a function of the SNR (see for example [16]). For SNRs larger than 50 dB, the error tended to reach 10 dBmm (10 mm in linear scale). 

Finally, the theoretical limits mentioned in Section 2.1 were evaluated for the specific case we simulated above. The first term in Equation (2) gave 53 ns (for *R*_0_ = 7.98 m) that was surely much lower than the tone duration (10 μs), so the short-range condition was respected. The far-target condition was expressed by Equation (4), obtaining:

(15) $$t-t_{0}\;\ll \;\frac{R_{0}}{\tau g}\;=\;20.35\;s$$

where we considered *R*_0_ = 7.98 m and *τ* = 40 ms. This condition was surely well-verified for a fall of a few seconds. The further condition in Equation (12), applied to a falling target for *t* > *t_0_*, gave the following:

(16) $$t-t_{0}\;\gg \;\frac{\tau }{2}=20\;\mathrm{ms}$$

This condition too was surely always verified in the experimental set-up described above.

## 3. Results

### 3.1. Falling Corner Reflector

**Figure 10:**
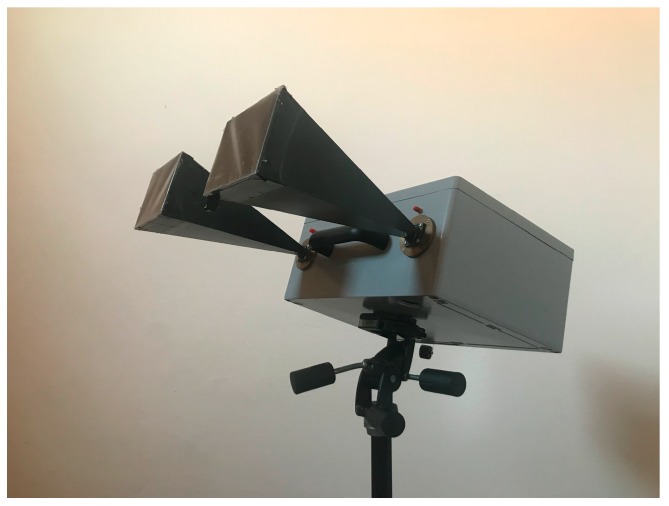
Picture of the radar prototype.

The SFCW radar used for the experimental tests was the prototype described in reference [17]. The radar is shown in Figure 10. It operates in K_u_ band with a maximum bandwidth of 380 MHz. The two antennas (transmitting and receiving) have a gain of 24 dBi and are vertically polarized. The transmitted power is 4 dBm. The minimum duration of a single tone is 10 μs.

**Figure 11:**
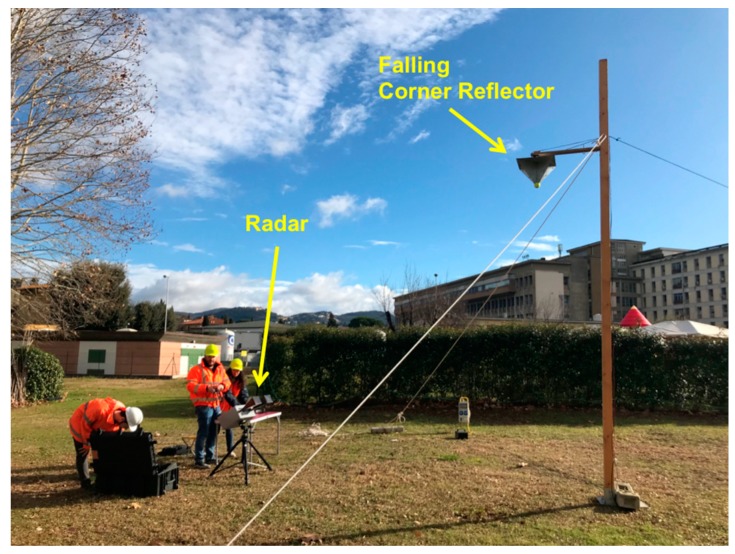
Picture of the experimental set-up for detecting a free-falling corner reflector by radar.

The radar was set with the same measurement parameters of the simulation described in the previous chapter. A corner reflector (CR) was fixed to an electromagnetic unhooking device that was put on the top of a pole 3.90 m high, as shown in Figure 11. The SFCW radar was pointed at the CR.

**Figure 12:**
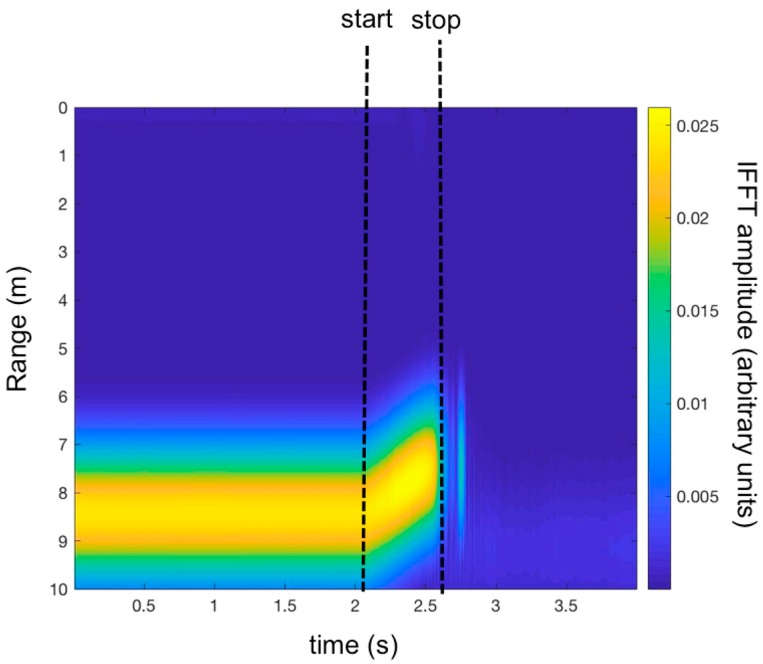
Time-range map of the measured IFFT (experimental set-up shown in Figure 8).

Figure 12 shows the map of the measured IFFT, with the time (a trace for each time sweep *τ*) along the *x*-axis, and the range, which was obtained by calculating the IFFT of each sweep, along the *y*-axis.

**Figure 13:**
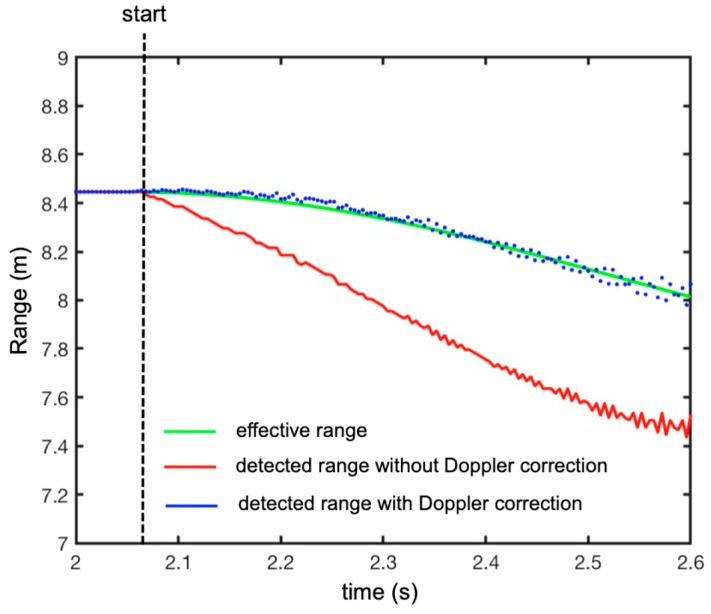
Measured plots of the peaks’ range in time (experimental set-up shown in Figure 8). The green line is the theoretical range of the target, the red line is the range of the peaks without the Doppler correction, and the blue dotted line is the range of the peaks with the Doppler correction.

Figure 13 shows the plot (in red) of the range of the peaks in time, the plot (in blue) of the peak with the Doppler correction, and the plot (in green) of the theoretical range of the free-falling target (by neglecting the friction with air).

**Figure 14:**
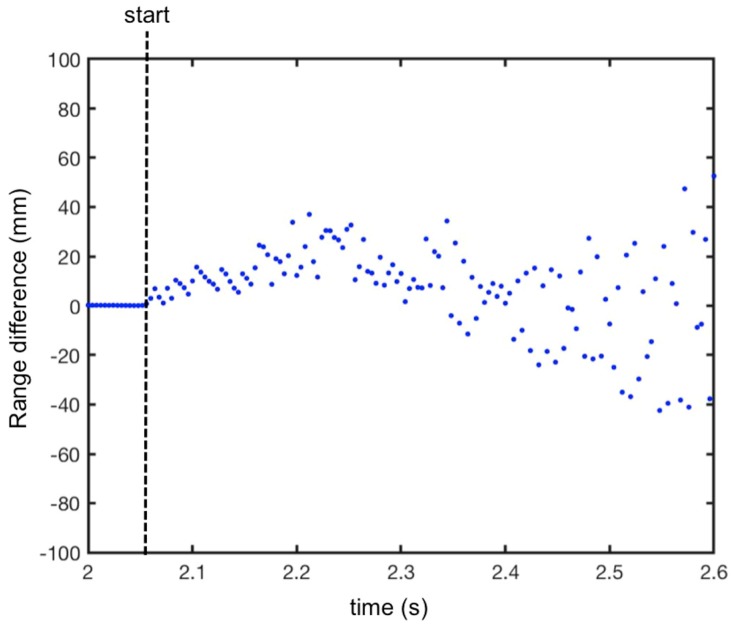
Difference between the effective range and the measured range with the Doppler correction during the fall.

In order to evaluate the accuracy of the technique, we calculated the difference between the effective range (green line in Figure 13) and the measured range with the Doppler correction (blue dotted line in Figure 13). The obtained plot is shown in Figure 14. As expected, the error increased with time because speed and acceleration increased with time. Nevertheless, the maximum error was about ±40 mm after 0.5 s of falling.

### 3.2. Detection of a Small UAV

**Figure 15:**
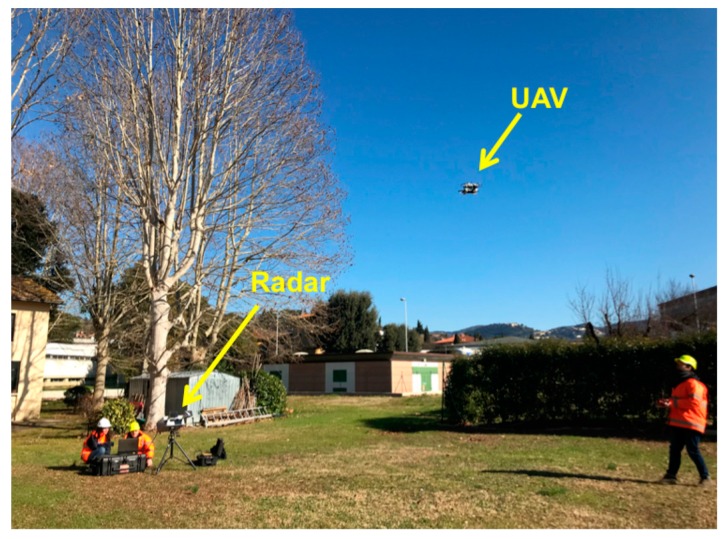
Detection of a small unmanned aerial vehicle (UAV) using an SFCW radar.

The experimental test described above was aimed to verify the technique in a fully controlled condition: position, speed, and acceleration of the free-falling CR were known at any instant of time. Furthermore, the target (i.e., the CR) was a body without moving parts and with a large radar cross section. In this experimental condition, the agreement between the effective and the detected range was very high. Therefore, the technique and the related algorithm were successfully validated. The next step was its application to the short-range detection of a small UAV, as shown in Figure 15.

As each tone has to be longer than the time of flight between the radar and the farthest target, by using a radar with 10 μs tone duration, the theoretical maximum range was 750 m, which could be a suitable range for this kind of application.

The radar equation [11] allowed us to estimate the SNR as:

(17) $$SNR=P_{0}\frac{G^{2}\sigma \tau }{{\left(4\pi \right)}^{2}R^{4}k_{B}T\left(NF\right)}$$

where *P_0_* is the transmitted power (4 dBm), *G* is the antenna gain (24 dBi), *σ* is the radar cross section of the drone (that was about 0.3 m^2^ [2]), *R* is the distance, *k_B_* is the Boltzmann’s constant, *T* is the temperature, and *NF* is the noise figure of the receiver (about 6 dB for the radar we used). As an example, at *R* = 1000 m, the *SNR* was 47 dB.

**Figure 16:**
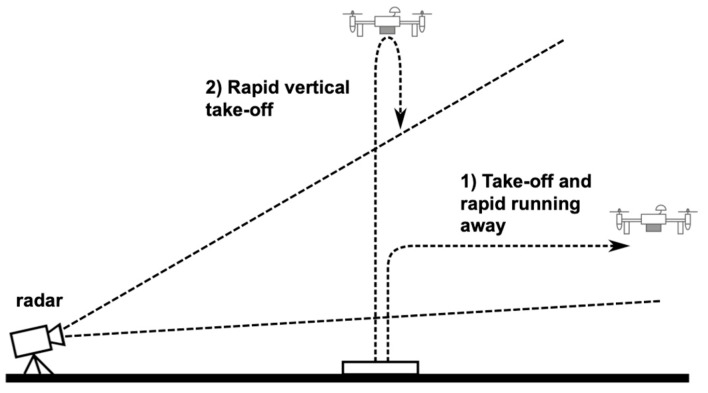
Two different flight maneuvers: (**1**) take-off and rapid running away and (**2**) rapid vertical take-off.

In order to test the radar technique in UAV detection, we performed radar measurements during two different flight maneuvers: (1) take-off and rapid running away and (2) rapid vertical take-off. Figure 16 shows a drawing of the two maneuvers. During each flight, the data of the accelerometers aboard of a UAV were recorded using telemetry.

**Figure 17:**
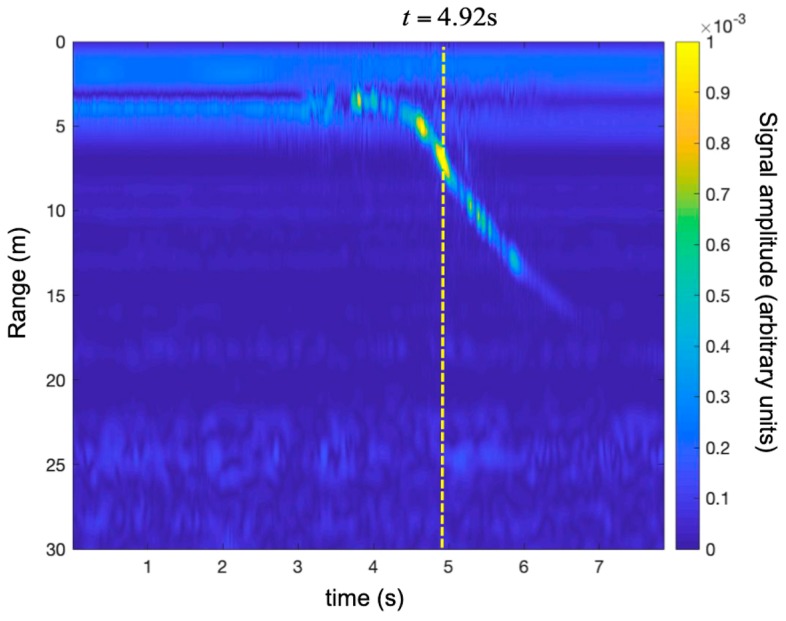
Detection of a small UAV using an SFCW radar. Time-range map without the Doppler correction during maneuver 1 (i.e., take-off and rapid running away).

**Figure 18:**
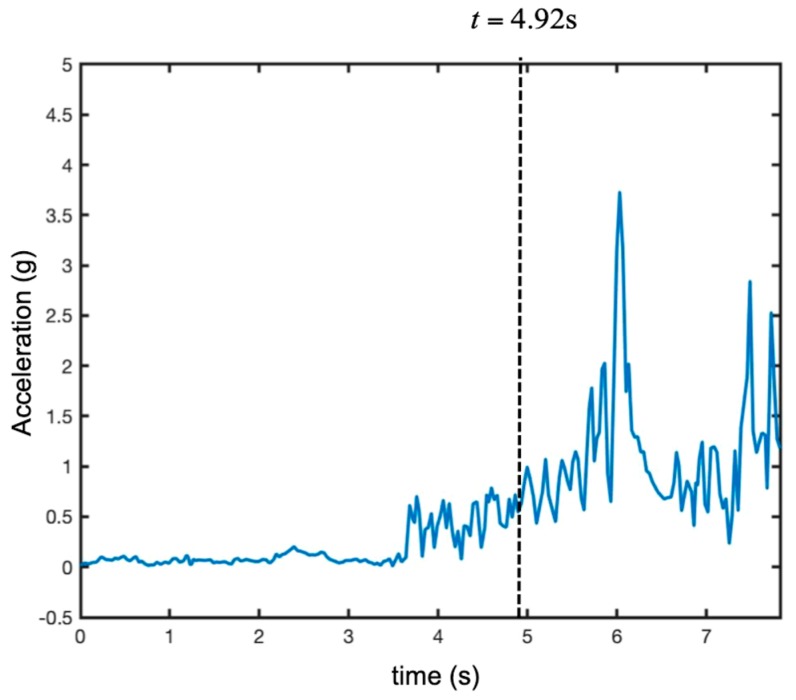
Recorded acceleration modulus of the UAV during maneuver 1 (i.e., take-off and rapid running away).

Figure 17 shows the time-range map acquired during maneuver 1. Figure 18 shows the recorded modulus of acceleration (i.e., the length of the acceleration vector). During the first 3.5 s, the UAV was stationary in the platform. At time 3.5 s, it took off and moved away from the radar at maximum speed. Its acceleration fluctuated rapidly around 1 g. At time 6.0 s, the drone slowed abruptly (the modulus of acceleration reached 3.5 g).

**Figure 19:**
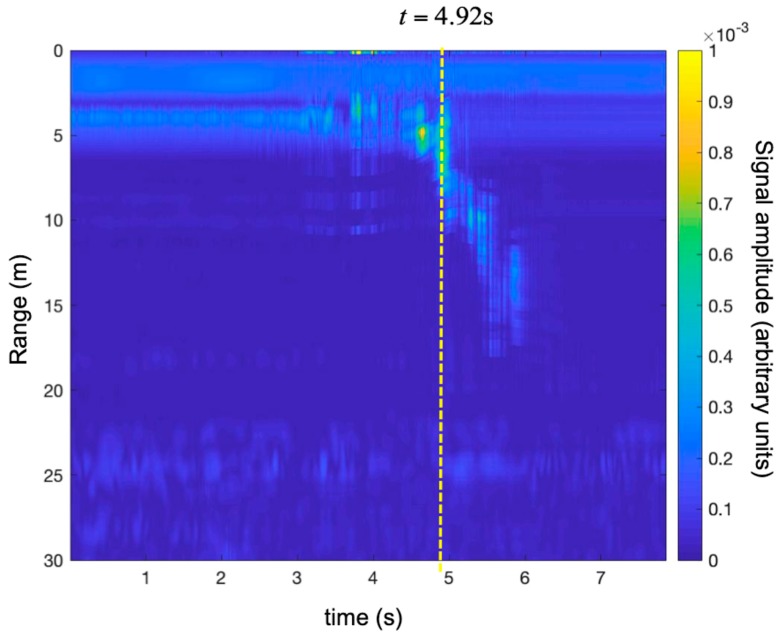
Detection of a small UAV using an SFCW radar. Time-range map with the Doppler correction during maneuver 1 (i.e., take-off and rapid running away).

Figure 19 shows the time-range map corrected using the technique described in Section 2.1.

**Figure 20:**
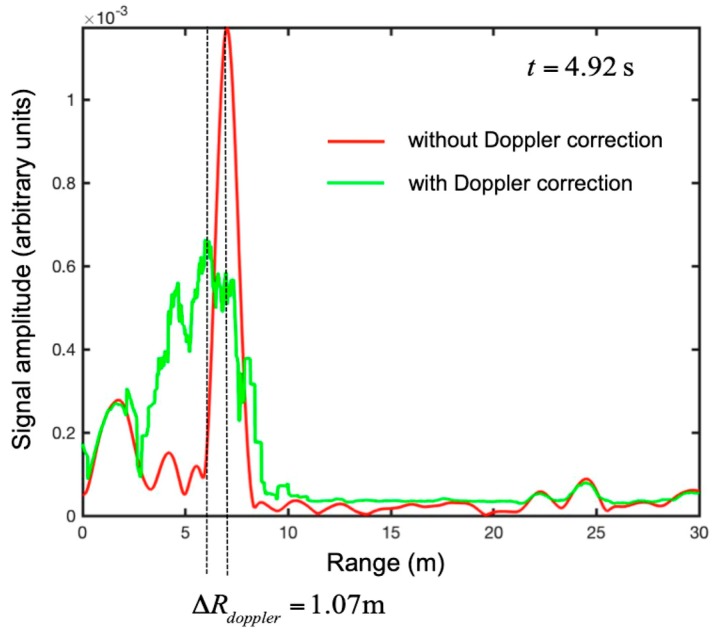
Maneuver 1 (i.e., take-off and rapid running away). Range plots at time *t* = 4.92 without the Doppler correction (**in red**) and with the Doppler correction (**in green**).

In order to appreciate the correction, we selected the traces at t = 4.92 s for both maps (with and without correction). The traces are shown in Figure 20. As expected, the correction shifted the peak 1.07 m back. This was consistent with the fact that when the target was moving away from the radar, the Doppler effect gave an overestimate of the range.

**Figure 21:**
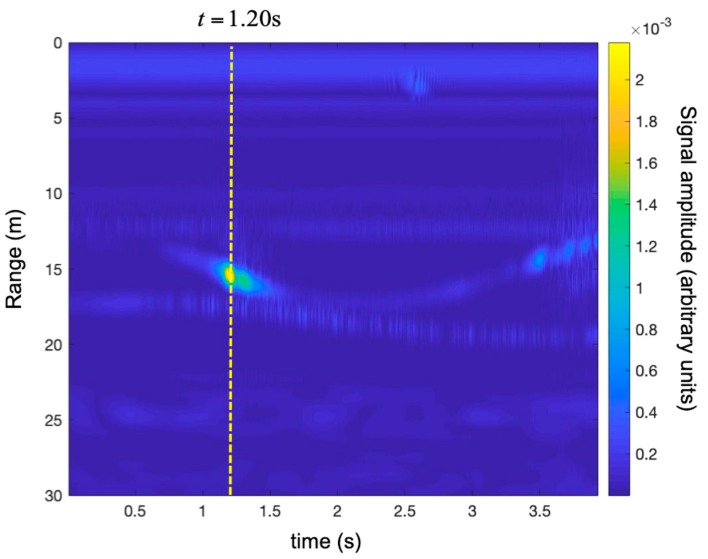
Detection of a small UAV using a SFCW radar. Time-range map without the Doppler correction during maneuver 2 (i.e., rapid take-off).

**Figure 22:**
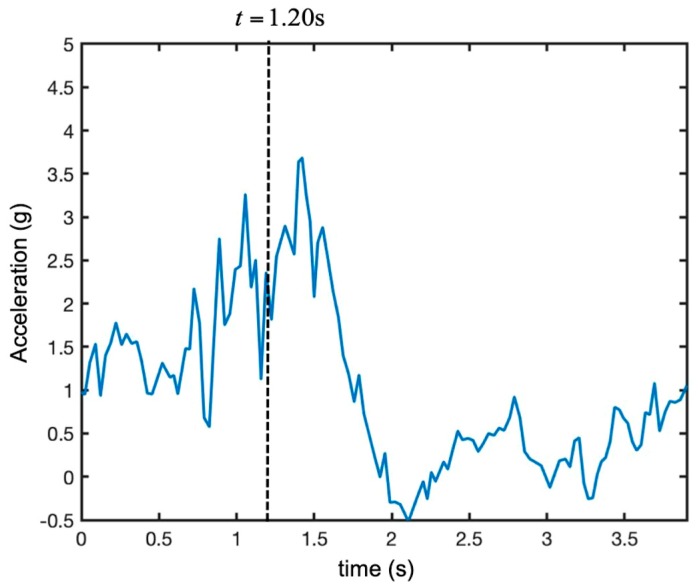
Recorded vertical acceleration of the UAV during maneuver 2 (i.e., rapid take-off).

The same radar measurements were performed during a different maneuver: a rapid take-off. Figure 21 shows the obtained time-range map. Figure 22 shows the recorded vertical acceleration. The UAV went up quickly with acceleration between 1 g and 3.5 g. At time 1.5 s, the motors were switched off, and the drone was free-falling for about 0.5 s. In the aftermath, the engines corrected the attitude before the landing. 

**Figure 23:**
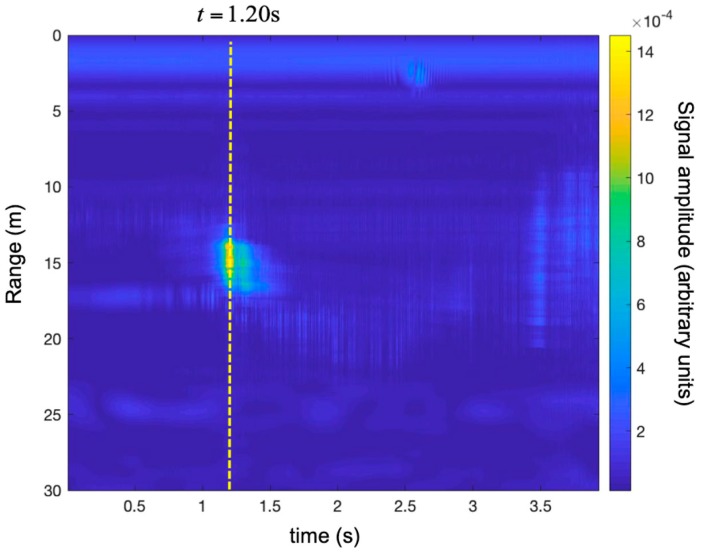
Detection of a small UAV using a SFCW radar. Time-range map with the Doppler correction during maneuver 2 (i.e., rapid take-off).

Figure 23 shows the time-range map corrected using the technique described in Section 2.1. 

**Figure 24:**
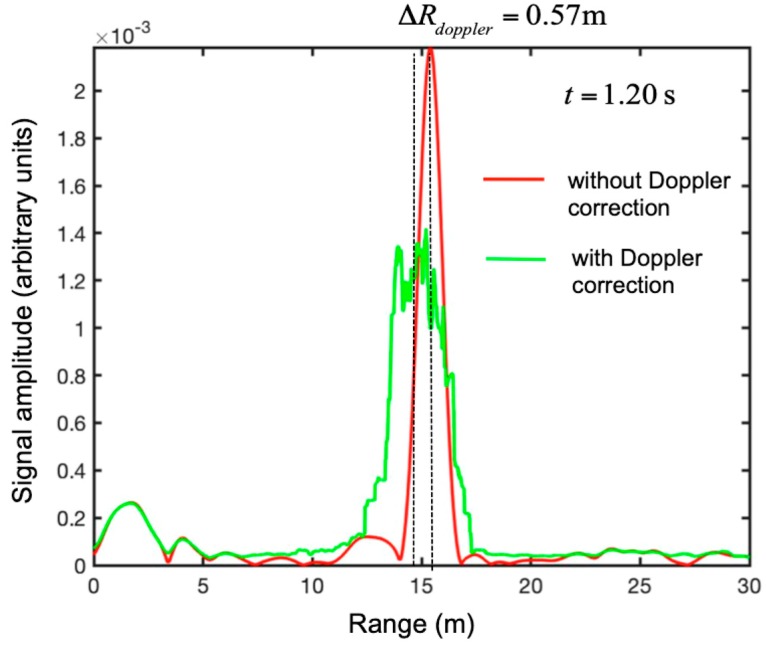
Maneuver 2 (i.e., rapid take-off). Range plots at time 1.20 s without the Doppler correction (**in red**) and with the Doppler correction (**in green**).

Figure 24 shows the comparison between the two traces at time *t* = 1.20 s. As expected, the corrected trace showed a peak closer to the radar (as the drone was moving away from the radar). The range shift was about 0.57 m.

Other measurements performed during different maneuvers gave similar results. For all tests, we noticed the application of the range compensation produced a sort of spreading of the target. This was probably due to the motion of the blades, as already discussed by the same authors of this article [10]. This is an aspect that has to be considered in the application of this correction. The point is whether it is worth correcting this drift when its correction produces a notable worsening of the radar’s images.

## 4. Conclusions

In this article, a Doppler compensation method for an SFCW radar is proposed and tested. In the controlled test with a corner reflector falling from the tip of a pole, the method was able to accurately retrieve the effective motion of the target. Nevertheless, in less controlled tests, the quality of the time-range map with the Doppler correction resulted lower than that of the original maps. Indeed, in real scenarios, the detected phase of the moving target can easily deteriorate by noise or spurious movements, and this can be an important limitation of this technique in real operative scenarios.

With respect to other Doppler range compensation techniques [13,14] that require an implementation of special modulation patterns, this technique can be used with any SFCW radar. On the other hand, the main disadvantage is probably the possible deterioration of the corrected image, as mentioned before. 

The Doppler compensation technique described in this article was specifically developed for UAV detection by an SFCW radar, but its validity is rather general and it could also be used, for example, for radar detection of rotating wind turbines [18], as well as in any application where it is necessary to track a target using an SFCW radar.
